# Rural-to-urban migration, socio-economic status and cardiovascular diseases risk factors among Bangladeshi adults: A nationwide population based survey

**DOI:** 10.3389/fpubh.2023.860927

**Published:** 2023-04-06

**Authors:** Shirin Jahan Mumu, Fiona F. Stanaway, Dafna Merom

**Affiliations:** ^1^School of Health Sciences, Western Sydney University, Sydney, NSW, Australia; ^2^School of Health Sciences, Torrens University Australia, Sydney, NSW, Australia; ^3^School of Public Health, University of Sydney, Sydney, NSW, Australia

**Keywords:** rural-to-urban migration, cardiovascular disease risk factors, Bangladesh, Urban Health Survey, obesity, rural, urban

## Abstract

**Background:**

Rural-to-urban migration is one of the key drivers of urbanization in Bangladesh and may impact on cardiovascular diseases (CVD) risk due to lifestyle changes. This study examined whether CVD risk factors were associated with migration to and duration of urban life, considering socio-economic indicators.

**Methods:**

A total of 27,792 participants (18–59 years) from the 2006 Bangladesh cross-sectional Urban Health Survey were included in the analyses of whom 14,167 (M: 7,278; W: 6,889) were non-migrant urban residents and 13,625 (M: 6,413; W: 7,212) were rural-to-urban migrants. Gender-specific prevalence of CVD risk factors were estimated for urban and migrant groups. Multivariate logistic regression models were used to test the association between each CVD risk by education and wealth within each study group and their possible effect modification. An analysis on the rural-to-urban migrant subgroup only was conducted to examine the association between each CVD risk factor and length of urban stay adjusted for demographic and socio-economic indicators.

**Results:**

Compared to urban residents, migrants had significantly lower prevalence of overweight/obesity for both genders. Hypertension was higher among urban women while alcohol/illicit drug use was higher among urban men. Mental health disorders were higher among migrants than urban residents for both genders and no difference were noted for diabetes or cigarette smoking prevalence. In both study groups and genders, the risk of overweight/obesity, hypertension and diabetes increased with increasing education and wealth whereas for mental health disorders, alcohol/illicit drug use, cigarette and bidi smoking the reverse was found. Differences in BMI between migrant and urban women were attenuated with increased education levels (*p* = 0.014 for interaction). Consistent increasing pattern of risk was observed with longer duration of urban stay; in migrant men for obesity (OR = 1.67), smoking (OR = 1.67) and alcohol/illicit drug use (OR = 2.86), and for obesity and mental health disorder among migrant women.

**Conclusions:**

Migrants had high proportion of CVD risk factors which were influenced by education, wealth and duration of urban stay.

## 1. Background

The rise of cardiovascular diseases (CVDs) in developing countries has been linked to progressive urbanization (1–3). Cross-sectional studies on comparisons of urban and rural areas have demonstrated higher rates of CVD risk factors for urban individuals (4–7). However, this comparison only suggests that the urban population is at higher risk of CVD, but does not give insights as to how these risks develop over time (8). Urban migration studies offer good opportunities to understand the contribution of environmental exposure in the progression of cardiometabolic risk over time. Initially urban migrants are healthier than the urban host population however, with increasing length of residence, migrants are acculturated to the urban diet and other lifestyles (e.g., physical inactivity) which increase the risk of CVD (9–11). Some studies on rural-to-urban migration have also demonstrated that body fat increases rapidly just after migration, whereas other cardiometabolic risk factors evolve gradually (12, 13). However, this process differs from population to population, as the relationship between environment, acculturation and health status is complex (14–17).

Socio-economic indicators play an important role in determining health in general (18) and has direct implication to CVD. The theory of cardiovascular epidemiologic transition states that risk factors and disease burden are initially higher in the affluent group, as they are the first who can afford products that are associated with unhealthy behaviors (e.g., cars, TV, and eating out) (19). However, these unhealthy behaviors are eventually adopted by the lower social classes, while the early adopters reduce their risk in response to preventive strategies (for example, weight management, smoking cessation programs and effective blood pressure control) (3, 19). Research has shown that most of the migration held due to the economic reason, therefore it is generally expected that a socio-economic gradient in health will eventually emerge in migrant group (20, 21), including in CVD risk. In the Indian Migration Study (IMS), evidence of effect modification by socio-economic status (SES) was found for the test of linear association between urban life years and percentage of body fat and the change in adiposity appeared in migrant from low SES position (12). A systematic review of acculturation and obesity showed that when people migrated from low-to-middle income countries to high income countries, the risk of overweight/obesity increased (22).

Urbanization is proceeding rapidly in Bangladesh. The percentage of urban growth has been estimated at about 3.5% per year (23) and the rural to urban migration rate was 4.29 per 1,000 persons per year for the whole country (24). As urbanization in Bangladesh is happening so rapidly, partly through migration from rural to urban areas, an improved understanding of its impact on cardiovascular risks after migration is paramount. CVD is an increasingly important cause of morbidity and mortality in Bangladesh with 27% of all deaths are attributed to CVD (25).

Despite the large and fast movement of rural residents toward urban areas in Bangladesh, to the best of our knowledge, no study has been conducted to date on rural-to-urban migrants' health status. Most of the studies on migration in Bangladesh have focused on reasons or determinants of migration (26, 27), socio-economic characteristics of migrants (28, 29) or reproductive health (30). This study therefore aimed to examine the association between CVD risk factors and duration of urban life after migration, considering SES as possible modifier. In doing so, this study may help to improve knowledge of transitions of chronic disease and identify high risk populations in Bangladesh to better inform non-communicable disease control.

## 2. Methods

### 2.1. Ethics approval

This secondary data-analysis study was approved by the Western Sydney University Human Research Ethics Committee (HREC # H11056). We obtained data following approval from the University of North Carolina at Chapel Hill (UNC), the data custodian. Informed consent was obtained from all subjects during data collection.

### 2.2. Data source

This study uses the 2006 Bangladesh Urban Health Survey (UHS) which is a cross-sectional nationally representative urban population sample. It was implemented through a collaborative effort of the National Institute of Population Research and Training (NIPORT) and Measure Evaluation, UNC, USA, Associates for Community and Population Research and funded by the United States Agency for International Development (USAID)/Bangladesh. The survey data was downloaded from the UNC's Dataverse (31).

### 2.3. Data collection method

A basic household-level questionnaire (32) usually administered to the female head which included a full roster of household members. Within each household, eligible individuals who were married and aged 10–59 years and all other adults aged 18–59 years were interviewed face-to-face for detailed information. Additional physical measurements were carried following standard methods. These measurements included height and weight, blood pressure (BP) using a mercury blood pressure machine, and fasting blood glucose (FBG) using the HemoCue Glucose 201+ in whole blood obtained by finger prick from capillaries in the middle or ring finger of the left arm after an overnight fasting in a seated position. BP and FBG were measured in randomly selected subsamples of adults over age 35. District Municipalities were not included for anthropometry, BP and FBG measurements. Fieldwork was carried out by 17 trained interviewing teams.

### 2.4. Study variables

#### 2.4.1. Exposure variables

We defined rural-to-urban migrants as those who had lived in a rural area prior to living in an urban area based on the response to “place of prior residence” (2006 UHS Men and women questionnaire—question no. 128). Non-migrant urban respondents were defined as those who had always lived in an urban environment based on two responses; either they had “always lived” (2006 UHS Men and women questionnaire—question no. 127) in their current place of residence or their “prior place of residence” was also an urban area (excluding those who had lived abroad). Here rural means village and urban includes city corporation with slum and non-slum and district municipalities.

The number of years the migrant had lived in an urban area was considered as a proxy for potential “acculturation” and was derived from the question “how long have you been living continuously in your current place of residence” (2006 UHS Men and women questionnaire—question no. 129). The number of years lived in an urban setting was categorized into quartiles. Respondents were also asked their main reasons for moving to the current place and we categorized into two broad categories which were; work or education and familial reasons.

#### 2.4.2. CVD risk factors

A current smoker was defined as having smoked either cigarettes or bidi (a small hand-rolled cigarette made of low grade unrefined tobacco flakes) within the last 1 month. Alcohol/illicit drug use was reported as participants who had ever used alcohol and/or illicit drug. UHS respondents were asked if they had ever used alcohol and/or illicit drugs in a single double-barrelled question. Thus, we use the term “alcohol/illicit drug use” throughout the paper.

BMI was calculated from weight and height and overweight and obesity were categorized using Asian cut-off values (33) for underweight (< 18.5 kg/m^2^), normal (18.5–23.0 kg/m^2^), overweight (23–27.5 kg/m^2^), and obesity (>27.5 kg/m^2^). Hypertension was defined as a systolic blood pressure ≥140 mmHg or a diastolic blood pressure ≥90 mmHg, or current treatment with an antihypertensive medication (32). Based on World Health Organization (WHO) guidelines, Impaired Fasting Glucose (IFG) was defined as a fasting blood glucose level of 6.1 mmol/L to 6.9 mmol/L and diabetes was defined as a level ≥7.0 mmol/L or self-reported diabetes medication use (34).

Mental health disorders were measured by a 20 item Self-Reporting Questionnaire (SRQ20) developed by WHO particularly for developing countries (35). SRQ20 is not a clinical diagnostic tool but it can screen for probable mental health disorders such as depression, anxiety and somatoform disorders among individuals (36). It is composed of 20 questions with yes (score of 1) or no (score of 0) responses and the maximum score is 20. We used the cut-off value of ≥8 for a probable case (37).

### 2.5. Statistical analysis

The gender-specific prevalence of CVD risk factors was estimated for the rural-to-urban migrant and urban non-migrant groups. The χ^2^ test was used to compare proportions of CVD risk factors between migrant and non-migrant and a *p*-value < 0.05 was considered statistically significant. The role of SES in explaining the association between migration status and CVD risk factor prevalence was further tested by comparing unadjusted and Mantel-Haenszel adjusted OR estimates (Table S1).

To examine the association between SES indicators and CVD risk factors, multiple logistic regression model was applied with each of the seven CVD risk factors as outcome and SES as independent variables while controlling for age. Each CVD risk factors were categorized as dichotomous (Yes/No), according to established cut points (see above for definitions). SES was evaluated by two independent variables: education level and household wealth quintile. Odds ratios (OR) and 95% confidence intervals (CI) were calculated for men and women and for migrants and urban non-migrants. Interaction effects of SES and migration status on each CVD risk factor were assessed by the type 3 Wald chi-square statistics in logistic regression models.

Thereafter, an analysis on the rural-to-urban migrant subgroup only was conducted to examine the association between each CVD risk factor and length of urban stay adjusted for age, education, marital status, employment status, domain, division and SES. Confidence interval (CI) of OR was calculated to get information about statistical significance, as well as the direction and strength of the effect. *P*-values were also included. Test for trend was determined by the significance of the continuous duration variable in the logistic regression model.

Regression analysis for the risk factors of smoking and alcohol/illicit drug use was not conducted for women because only eight women reported smoking and five alcohol/illicit drug consumption. All analyses were stratified by gender. Data analyses were performed using SPSS version 23.0 (SPSS Inc, Chicago, IL).

## 3. Results

### 3.1. Study population characteristics and migration status

Table 1 shows the socio-demographic characteristics of all respondents by migration status. A total of 27,792 participants (51% females) were included in the analyses, after excluding 218 individuals whose previous place of residence was abroad. Overall, 14,167 (M: 7,278; W: 6,889) were non-migrant urban residents and 13,625 (49%, M: 6,413; W: 7,212) were rural-to-urban migrants. Rural-to-urban migrant men and women were slightly older than their non-migrant counterparts (34.44 ± 11.02 vs. 32.80 ± 10.85; *p* ≤ 0.001). Non-migrant urban residents were more likely to have attended school than migrants. Among females, 60.9% migrants had attended school, compared to 72.9% of non-migrants (*p* < 0.001). Most men were currently employed but the proportion was higher for migrants than urban residents (93.4% vs. 88.1%; *p* < 0.001). Women were far less likely than men to be currently employed and female employment was lower in non-migrant than in migrant women (24.0% vs. 34.6%; *p* < 0.001). More than half of the migrants lived in a slum neighborhood in City Corporation (M: 56.2%; W: 55.0%) whereas the majority of urban non-migrants were living in a non-slum neighborhood in City Corporation (M: 43.7%; W: 42.7%).

**Table 1:** Study population characteristics by gender and place of origin.

**Study population characteristics**	**Men (*****n*** = **13,691)**		***p***	**Women (*****n*** = **14,101)**		***p***
	**Urban non-migrants**	**Rural-to-urban migrants**		**Urban non-migrant**	**Rural-to-urban migrants**	
***n*** **(%)**	**7,278 (53.2)**	**6,413 (46.8)**		**6,889 (48.9)**	**7,212 (51.1)**	
Age (year)						
≤ 20	922 (12.7)	680 (10.6)	< 0.001	1,296 (18.8)	1,237 (17.2)	0.004
21–30	2,751 (37.8)	2,125 (33.1)		2,526 (36.7)	2,570 (35.6)	
31–40	1,755 (24.1)	1,607 (25.1)		1,716 (24.9)	1,952 (27.1)	
>40	1,850 (25.4)	2,001 (31.2)		1,351 (19.6)	1,453 (20.1)	
Ever attended school	5,943 (81.7)	4,596 (71.7)	< 0.001	5,020 (72.9)	4,392 (60.9)	< 0.001
Highest grade of education						
Primary level	1,538 (21.1)	1,565 (24.4)	< 0.001	1,586 (23.0)	1,922 (26.7)	< 0.001
High school level	3,512 (48.3)	2,505 (39.1)		2,981 (43.3)	2,281 (31.6)	
University level	893 (12.3)	526 (8.2)		453 (6.6)	189 (2.6)	
Marital status						
Currently married	4,750 (65.3)	4,932 (76.9)	< 0.001	5,153 (74.8)	5,980 (82.9)	< 0.001
Never married	2,479 (34.1)	1,450 (22.6)		1,063 (15.4)	465 (6.4)	
Others (separated/divorced/ descended/widow)	49 (0.7)	31 (0.5)		673 (9.8)	767 (10.6)	
Muslim religion	6,462 (88.8)	5,807 (90.6)	< 0.001	6,289 (91.3)	6,506 (90.2)	0.015
Currently employed	6,411 (88.1)	5,991 (93.4)	< 0.001	1,653 (24.0)	2,497 (34.6)	< 0.001
Domains						
Slum neighborhood in City Corporation	2,838 (39.0)	3,602 (56.2)	< 0.001	2,808 (40.8)	3,965 (55.0)	< 0.001
Non-slum neighborhood in City Corporation	3,181 (43.7)	2,420 (37.7)		2,945 (42.7)	2,561 (35.5)	
District municipalities	1,259 (17.3)	391 (6.1)		1,136 (16.5)	686 (9.5)	
Household wealth quintile						
Q1 (poorest)	1,275 (17.5)	1,796 (28.0)	< 0.001	1,244 (18.1)	2,207 (30.6)	< 0.001
Q2	1,318 (18.1)	1,452 (22.6)		1,208 (17.5)	1,599 (22.2)	
Q3	1,412 (19.4)	1,262 (19.7)		1,303 (18.9)	1,243 (17.2)	
Q4	1,783 (24.5)	1,070 (16.7)		1,575 (22.9)	1,148 (15.9)	
Q5 (richest)	1,490 (20.5)	833 (13.0)		1,559 (22.6)	1,015 (14.1)	
Total income during last month of employed, quartile						
Q1	1,651 (26.4)	1,431 (24.1)	< 0.001	508 (31.4)	609 (24.7)	< 0.001
Q2	1,440 (23.1)	1,586 (26.7)		337 (20.8)	607 (24.6)	
Q3	1,423 (22.8)	1,616 (27.2)		316 (19.5)	700 (28.4)	
Q4	1,730 (27.7)	1,308 (22.0)		457 (28.2)	547 (22.2)	

Results are expressed as number (%); X^2^ test was performed.

Among men, half of the migrants (50.6%) were in two lowest household wealth quintiles. In contrast nearly half (45%) of the urban residents were in the two wealthiest quintiles. Almost equal proportion (≈19%) of migrant and urban residents were in the middle (or third) quintile. A nearly similar distribution was found for women. For monthly income, 27.7% urban men were in the highest quartile whereas 22.0% of migrants were in the highest quartile. This pattern was similar for women.

### 3.2. CVD risk factors and migration status

Table 2 presents the distribution of CVD risk factors by migration status among men and women. Bidi smoking was higher in rural-to-urban migrants (10.7% vs. 5.6%; *p* < 0.001) but a lower prevalence of alcohol/illicit drug use was found in migrants than in non-migrants urban residents (9.5% vs. 14.7%; *p* < 0.001).

**Table 2:** Gender-specific prevalence of cardiovascular disease risk factors by migration status.

**Risk factors**	**Men (*****n*** = **13,691)**		***p***	**Women (*****n*** = **14,101)**		***p***
	**Urban non-migrants**	**Rural-to-urban migrants**		**Urban non-migrants**	**Rural-to-urban migrants**	
**Smoking and alcohol/illicit drug use**, ***n*** **(%)**	***n*** **=** **7,278**	***n*** **=** **6,413**		***n*** **=** **1,063**	***n*** **=** **465**	
Cigarette smoking in last 1 month	3,590 (49.3)	3,110 (48.5)	0.17	8 (0.1)	0 (0)	–
Bidi smoking in last 1 m	411 (5.6)	685 (10.7)	< 0.001	0 (0)	0 (0)	–
Ever used alcohol/illicit drug	1,068 (14.7)	609 (9.5)	< 0.001	4 (0.1)	1 (0.1)	–
**Body mass index (BMI)**, ***n*** **(%)**	***n*** **=** **3,346**	***n*** **=** **3,246**		***n*** **=** **3,135**	***n*** **=** **3,539**	
Underweight	875 (26.2)	945 (29.1)	< 0.001	533 (17.0)	814 (23.0)	< 0.001
Normal weight	1,522 (45.5)	1,628 (50.2)		1,261 (40.2)	1,553 (43.9)	
Overweight	450 (13.4)	335 (10.3)		427 (13.6)	408 (11.5)	
Obesity	499 (14.9)	338 (10.4)		914 (29.2)	764 (21.6)	
**Blood glucose**, ***n*** **(%)**	***n*** **=** **515**	***n*** **=** **641**		***n*** **=** **445**	***n*** **=** **644**	
IFG	30 (5.8)	29 (4.5)	0.60	21 (4.7)	29 (4.5)	0.26
Diabetes	56 (10.9)	69 (10.8)		59 (13.3)	65 (10.1)	
**Blood pressure**, ***n*** **(%)**	***n*** **=** **597**	***n*** **=** **762**		***n*** **=** **499**	***n*** **=** **706**	
Hypertension	212 (35.5)	249 (32.7)	0.29	231 (46.3)	282 (39.9)	0.016
**Mental health disorder**, ***n*** **(%)**	***n*** **=** **7,278**	***n*** **=** **6,413**		***n*** **=** **6,889**	***n*** **=** **7,212**	
Probable case	1,226 (16.8)	1,226 (19.1)	< 0.001	2,266 (32.9)	2,587 (35.9)	< 0.001

Results are expressed as number (%); X^2^ was performed. The bold values indicate the sample size.

Undernutrition (BMI 18.5) was more common among rural-to-urban migrants (M: 29.1%; W: 23%) than non-migrant urban residents (M: 10.3%; W: 17.4%). By contrast, overweight and obesity was significantly higher in the non-migrant urban group with the highest prevalence in non-migrant urban women (42.8%) and the lowest prevalence in migrant men (20.7%). Diabetes prevalence was comparable between migrant and non-migrant urban men (10.9% vs. 10.8%: respectively). However, in women, diabetes was more common in non-migrant urban residents (13.3%) than migrants (10.1%); though, the difference did not reach statistical significance. Nearly half (46.3%) of urban non-migrant women and 39.9% of migrant women had hypertension (*p* = 0.016). Among men, the corresponding prevalence rates were lower and quite similar between the groups (35.5% for non-migrant urban and 32.7% for migrants; *p* = 0.29). The highest proportion of probable cases of mental health disorder was found among rural-to-urban migrant women (35.9%) and the lowest in non-migrant urban men (16.8%).

### 3.3. Socio-economic status, migration status and CVD risks

The gender-specific unadjusted associations between migration status, education level and household wealth and each CVD risk factor are presented in Table S1. Among men, in unadjusted analyses a statistically significant relationship was observed between migration status and obesity (OR = 0.66, 95% CI: 0.59–0.74), mental health disorder (OR = 1.17, 95% CI: 1.07–1.27), bidi smoking (OR = 1.99, 95% CI: 1.76–2.27) and alcohol/illicit drug use (OR = 0.61, 95% CI: 0.55–0.68). Among women, migration status was associated with obesity (OR = 0.66, 95% CI: 0.60–0.73), hypertension (OR = 0.77, 95% CI: 0.61–0.97), and mental health (OR = 1.14, 95% CI: 1.06–1.22) in unadjusted analyses. The Mantel Haenszel odds ratios (adjusted once for education and once for HH wealth) were calculated to estimate the percent changes to the crude associations. Among men, adjusting for education and household wealth changed the association more than ten percent between migration status and obesity and bidi smoking. In contrast in women, adjustment for these SES measures the associations are altered between migration status and obesity.

Table 3 shows that an SES gradient, adjusted for age, exists both in migrants and urban residents. The risk of overweight and obesity, hypertension and diabetes increased with increasing SES whereas the risk of mental health disorders, alcohol/illicit drug use, cigarette and bidi smoking decreased with increasing SES status, regardless of migration status in both men and women.

**Table 3:** Association between socio-economic status and CVDs risk factors among rural-to-urban migrant and urban resident.

**Risk factors**	**SES**	**Men**		**Women**	
		**Urban non-migrants OR (95% CI);** ***p***	**Rural-to-urban migrant OR (95% CI);** ***p***	**Urban non-migrants OR (95% CI);** ***p***	**Rural-to-urban migrant OR (95% CI);** ***p***
Overweight and obesity	**Education** ^a^				
	Illiterate	Ref	Ref	Ref	Ref
	Primary	1.24 (0.89–1.74); 0.202	1.40 (0.99–1.97); 0.05	1.54 (1.22–1.96); 0.001	1.44 (1.17–1.79); 0.001
	High school and above	2.46 (1.83–3.31); 0.001	2.94 (2.15–4.02); 0.001	1.54 (1.21–1.96); 0.001	1.68 (1.33–2.13); 0.001
	**HH wealth quintile** ^b^				
	Q1 (poorest)	Ref	Ref	Ref	Ref
	Q2	1.08 (0.72–1.61); 0.704	1.71 (1.17–2.51); 0.006	1.47 (1.08–2.01); 0.016	1.38 (1.07–1.78); 0.01
	Q3	2.13 (1.50–3.04); 0.001	2.89 (2.01–4.16); 0.001	2.40 (1.79–3.21); 0.001	3.21 (2.50–4.12); 0.001
	Q4	3.65 (2.60–5.13); 0.001	5.76 (4.00–8.30); 0.001	3.49 (2.60–4.69); 0.001	4.15 (3.16–5.44); 0.001
	Q5 (richest)	5.55 (3.91–7.87); 0.001	9.49 (6.52–13.82); 0.001	6.11 (4.47–8.35); 0.001	7.60 (5.70–10.15); 0.001
Hypertension	**Education** ^**a**^				
	Illiterate	Ref	Ref	Ref	Ref
	Primary	0.99 (0.57–1.74); 0.99	1.58 (1.01–2.49); 0.047	1.60 (0.95–2.69); 0.075	0.86 (0.56–1.34); 0.51
	High school and above	1.26 (0.75–2.10); 0.38	2.28 (1.42–3.66); 0.001	1.28 (0.74–2.22); 0.384	0.74 (0.44–1.24); 0.25
	**HH wealth quintile** ^b^				
	Q1 (poorest)	Ref	Ref	Ref	Ref
	Q2	1.12 (0.50–2.52); 0.78	1.23 (0.73–2.08); 0.44	0.66 (0.29–1.51); 0.323	1.06 (0.63–1.79); 0.83
	Q3	2.59 (1.24–5.41); 0.01	1.73 (1.03–2.91); 0.037	0.83 (0.38–1.82); 0.645	1.76 (1.04–2.96); 0.03
	Q4	3.02 (1.47–6.19); 0.003	1.56 (0.89–2.74); 0.124	1.60 (0.77–3.32); 0.213	4.05 (2.31–7.08); 0.001
	Q5 (richest)	2.96 (1.40–6.23); 0.004	1.72 (0.98–3.03); 0.058	2.13 (0.96–4.72); 0.062	4.04 (2.26–7.24); 0.001
Diabetes	**Education** ^a^				
	Illiterate	Ref	Ref	Ref	Ref
	Primary	0.84 (0.24–2.96); 0.79	0.98 (0.37–2.64); 0.97	2.14 (0.88–5.21); 0.093	0.68 (0.31–1.52); 0.35
	High school and above	2.00 (0.73–5.52); 0.18	2.30 (0.93–5.70); 0.072	1.46 (0.60–3.57); 0.402	1.04 (0.47–2.27); 0.93
	**HH wealth quintile** ^b^				
	Q1 (poorest)	Ref	Ref	Ref	Ref
	Q2	1.04 (0.16–6.59); 0.97	1.45 (0.47–4.49); 0.52	0 (0–0)	1.76 (0.46–6.74); 0.41
	Q3	1.20 (0.20–7.06); 0.84	1.80 (0.59–5.50); 0.304	2.14 (0.23–20.17); 0.51	4.79 (1.46–15.65); 0.009
	Q4	3.19 (0.65–15.63); 0.15	2.25 (0.74–6.82); 0.153	4.92 (0.61–39.84); 0.14	3.88 (1.09–13.74); 0.036
	Q5 (richest)	3.62 (0.73–18.03); 0.12	3.60 (1.24–10.41); 0.018	8.60 (1.04–71.43); 0.046	10.48 (3.14–34.90); 0.001
Mental health disorder	**Education** ^a^				
	Illiterate	Ref	Ref	Ref	Ref
	Primary	1.01 (0.84–1.20); 0.94	0.86 (0.73–1.01); 0.06	0.83 (0.72–0.96); 0.011	0.93 (0.82–1.06); 0.28
	High school and above	0.72 (0.60–0.86); 0.001	0.69 (0.58–0.82); 0.001	0.64 (0.55–0.75); 0.001	0.64 (0.55–0.74); 0.001
	**HH wealth quintile** ^b^				
	Q1 (poorest)	Ref	Ref	Ref	Ref
	Q2	0.87 (0.72–1.04); 0.13	0.73 (0.62–0.87); 0.001	0.93 (0.79–1.09); 0.36	0.86 (0.75–0.98); 0.025
	Q3	0.66 (0.54–0.80); 0.001	0.68 (0.56–0.82); 0.001	0.87 (0.74–1.03); 0.116	0.83 (0.72–0.97); 0.017
	Q4	0.50 (0.40–0.61); 0.001	0.53 (0.42–0.67); 0.001	0.58 (0.49–0.69); 0.001	0.55 (0.46–0.66); 0.001
	Q5 (richest)	0.42 (0.33–0.53); 0.001	0.42 (0.32–0.55); 0.001	0.43 (0.36–0.53); 0.001	0.43 (0.35–0.53); 0.001
Cigarette smoking	**Education** ^a^				
	Illiterate	Ref	Ref	–	–
	Primary	1.05 (0.90–1.22); 0.54	0.90 (0.79–1.04); 0.16		
	High school and above	0.88 (0.76–1.01); 0.07	0.54 (0.47–0.63); 0.001		
	**HH wealth quintile** ^b^				
	Q1 (poorest)	Ref	Ref		
	Q2	0.88 (0.75–1.03); 0.10	1.08 (0.93–1.24); 0.32		
	Q3	0.79 (0.66–0.91); 0.002	1.10 (0.94–1.28); 0.23	–	–
	Q4	0.64 (0.54–0.75); 0.001	0.71 (0.60–0.85); 0.001		
	Q5 (richest)	0.58 (0.49–0.69); 0.001	0.63 (0.52–0.76); 0.001		
Bidi smoking	**Education** ^a^				
	Illiterate	Ref	Ref	–	–
	Primary	0.65 (0.50–0.82); 0.001	0.58 (0.48–0.72); 0.001		
	High school and above	0.28 (0.21–0.38); 0.001	0.34 (0.26–0.44); 0.001		
	**HH wealth quintile** ^**b**^				
	Q1 (Poorest)	Ref	Ref		
	Q2	0.56 (0.44–0.73); 0.001	0.44 (0.36–0.54); 0.001		
	Q3	0.36 (0.27–0.49); 0.001	0.22 (0.16–0.29); 0.001	–	–
	Q4	0.13 (0.08–0.20); 0.001	0.08 (0.05–0.14); 0.001		
	Q5 (Richest)	0.02 (0.01–0.06); 0.001	0.02 (0.01–0.07); 0.001		
Alcohol/illicit drug use	**Education** ^**a**^				
	Illiterate	Ref	Ref	–	–
	Primary	0.89 (0.73–1.09); 0.26	1.15 (0.92–1.43); 0.22		
	High school and above	0.81 (0.67–0.98); 0.27	0.76 (0.59–0.96); 0.02		
	**HH wealth quintile** ^b^				
	Q1 (Poorest)	Ref	Ref		
	Q2	0.86 (0.70–1.06); 0.15	0.86 (0.68–1.07); 0.17		
	Q3	0.77 (0.62–0.95); 0.02	0.68 (0.53–0.88); 0.004	–	–
	Q4	0.65 (0.52–0.81); 0.001	0.69 (0.51–0.93); 0.014		
	Q5 (Richest)	0.72 (0.57–0.91); 0.001	0.82 (0.59–1.14); 0.25		

^a^Adjusted for current age and HH wealth quintile; ^b^Adjusted for current age and education.

Lifestyles were not assessed in women as few women reported these behaviors.

Adjusted R^2^ (range) = 5%−30% Overall model Chi-squared-test, p < 0.001.

**Figure 1:**
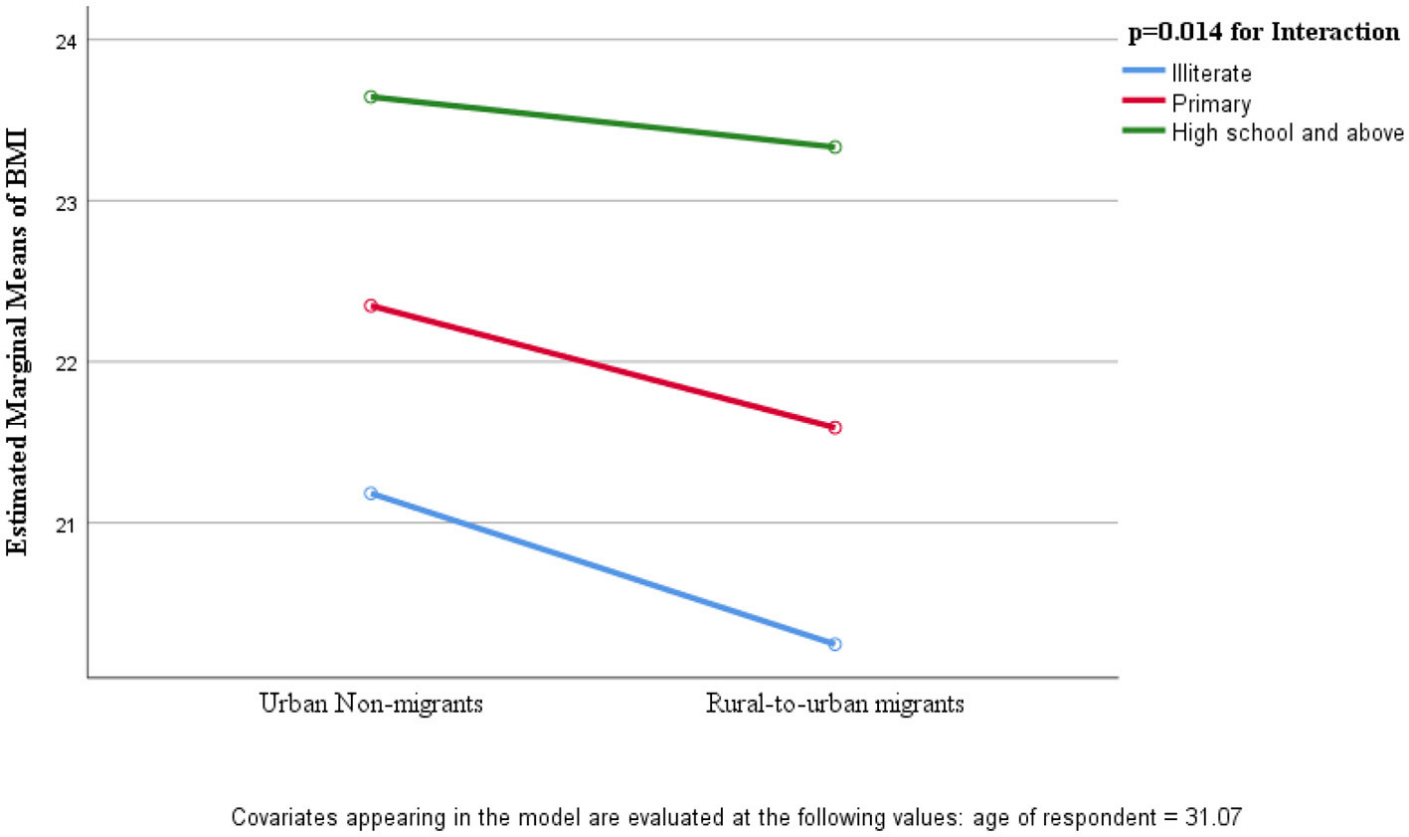
Mean BMI of women by education and migration status.

We have examined interactions between SES (i.e., education levels and household wealth quintiles) and migration status on CVD risk factors; generally, CVD risk factors appeared to be higher in rural-to-urban migrants than their counterparts, urban non migrants of the same SES status. While no significant interactions were observed in men, significant interactions in women were found between education and migration status on BMI (*p* = 0.014, *R*^2^ = 0.16, *F* = 4.27) (Figure 1). Among women from a high education level, the differences in BMI between rural-to-urban migrants and urban residents were negligible whereas among women from lower education levels, the differences in BMI were substantial.

### 3.4. CVD risk factors and migration indicators

Table 4 shows migration indicators associated with CVD risk factors among migrants only, adjusting for confounders mentioned in the method. A consistent increasing pattern of risk was observed with longer duration of urban stay in migrant men (*p* for trend = 0.007 for obesity and < 0.001 for cigarette smoking and alcohol/illicit drug use). In contrast, longer duration of stay was associated with decreasing risk of bidi smoking in men (*p* < 0.001). Among women, increased duration of stay was associated with increasing risk of overweight and obesity, and mental health disorder (*p* for trend < 0.001). In women, migrating for work or education (rather than any other reason) was associated with higher likelihood of hypertension, OR 2.07 (1.34, 3.18).

**Table 4:** Length of urban years and reasons for migration and their association with CVD risk factors among rural-to-urban migrant men and women.

**Variables**	**Risk factors**						
	**Overweight and obesity**	**Mental health disorder**	**Hypertension**	**Diabetes**	**Cigarette smoking**	**Bidi smoking**	**Alcohol/ illicit drug use**
**Adjusted OR (95% CI);** ***p***							
**Men (*****n*** = **6,413)**							
**Length of years lived**							
Q1 (≤ 5 years)	ref	ref	ref	ref	ref	ref	ref
Q2 (6–12 years)	1.20 (0.87–1.64); 0.27	1.02 (0.85–1.24); 0.82	1.26 (0.66–2.43); 0.47	0.82 (0.26–2.58); 0.74	1.15 (0.99–1.34); 0.06	0.68 (0.53–0.87); 0.002	1.47 (1.11–1.96); 0.008
Q3 (13–20 years)	1.32 (0.94–1.84); 0.11	0.98 (0.80–1.20); 0.86	1.10 (0.59–2.01); 0.77	0.55 (0.18–1.63); 0.28	1.40 (1.20–1.64); 0.001	0.54 (0.42–0.70); 0.001	2.21 (1.66–2.95); 0.001
Q4 (>20 years)	1.67 (1.15–2.41); 0.01	1.08 (0.86–1.36); 0.51	1.58 (0.88–2.82); 0.12	1.22 (0.47–3.16); 0.68	1.67 (1.40–2.02); 0.001	0.40 (0.29–0.53); 0.001	2.86 (2.04–4.00); 0.001
*p* for trend	0.007	0.66	0.13	0.45	< 0.001	< 0.001	< 0.001
**Reason for migration**							
Migration for family, marriage and other	ref	ref	ref	ref	ref	ref	ref
Migrated for work, employment and education	1.23 (0.91–1.65); 0.18	1.06 (0.87–1.29); 0.59	1.08 (0.66–1.77); 0.76	1.10 (0.49–2.46); 0.82	0.94 (0.80–1.09); 0.41	1.23 (0.93–1.64); 0.15	0.66 (0.53–0.83); 0.001
**Women (*****n*** = **7,212)**							
**Length of years lived**							
Q1 (≤ 4 years)	ref	ref	ref	ref			
Q2 (5–10 years)	1.20 (0.96–1.51); 0.11	0.99 (0.86–1.14); 0.88	1.59 (0.84–3.01), 0.15	0.52 (0.18–1.51); 0.23			
Q3 (11–18 years)	1.53 (1.20–1.95); 0.001	1.28 (1.10–1.49); 0.001	0.95 (0.53–1.71); 0.86	0.19 (0.06–0.62); 0.01	–	–	–
Q4 (>19 years)	1.74 (1.32–2.30); 0.001	1.35 (1.13–1.60); 0.001	1.40 (0.81–2.41); 0.23	0.74 (0.32–1.70); 0.48			
*p* for Trend	< 0.001	< 0.001	0.46	0.79			
**Reason for migration**							
Migration for family, marriage and other	ref	ref	ref	ref			
Migrated for work, employment and education	0.87 (0.71–1.10); 0.15	1.10 (0.96–1.23); 0.19	2.07 (1.34–3.18); 0.001	0.90 (0.41–1.97); 0.79	–	–	–

Adjusted for age, education, marital status, employment status, domain, division, SES.

Adjusted R^2^ (range) = 13%−32% Overall model Chi-squared-test, p < 0.001.

## 4. Discussion

In this study we looked at the relationship between internal migration from rural to urban areas and CVD risk factors and role of SES among the Bangladeshi population. Our main findings are the CVD risk profile of migrants was generally healthier than non-migrants in urban areas, with the exception of bidi smoking and mental health. Adjustment for differences in education and wealth between migrants and non-migrants attenuated many of the observed differences in CVD risk factors. Analyses in migrants only showed that most of the CVD risk factors tended to increase with a longer duration of residence in an urban area.

### 4.1. CVD risk factors differences between migrants and non-migrants in urban areas

Among all CVD risk factors, weight status presented the most marked differences between migrants and the urban group. Obesity and overweight was lower in migrants than urban residents, and the prevalence of underweight was higher among migrants than urban group suggesting that Bangladesh is facing a double burden of underweight and overweight. It is important to highlight that one-third and one-fourth of rural-to-urban migrant women and men, respectively, were overweight and obese, which is quite high.

Although we did not find significant differences in diabetes prevalence between migrants and the urban group in both genders, it is possible to expect increase in the burden of diabetes if weight gain is not prevented among rural–urban migrants. Relatively few studies have investigated the impact of rural-to-urban migration on glucose level (38–42). In the Indian migration study (IMS), fasting blood glucose levels among migrant and non-migrant urban groups (38) were not different, as in our study. In contrast, glucose levels were found to be lower in the migrant than the non-migrant urban group in Peru (39) and Poland (40). In the WHO Study on global AGEing and adult health (SAGE) study of six low-and-middle income countries, the prevalence of doctor diagnosed diabetes was significantly higher in non-migrant urban dwellers (RR 1.69, 95% CI: 1.15–2.47) followed by migrants, and then rural groups in the pooled analysis. In contrast, in country-specific analyses, prevalence was higher in migrants than non-migrant urban residents in Ghana and Russia (42). However, differences in the way diabetes was defined in these studies may challenge comparisons between countries in relation to these sub-groups. The prevalence of hypertension in our study was quite similar in both groups among men, whereas urban non-migrant women had a higher prevalence of hypertension than migrant women. In a systematic review on the effect of internal migration on CVD risk factors in low and middle income countries the prevalence of CVD risk factors in migrants were lower than the urban non-migrants for both genders (43).

Smoking behavior was more prevalent in migrants, mainly due to higher bidi smoking among migrants. This could be due to the low cost of bidi which makes it more affordable for migrants. Smoking is not a socially or culturally acceptable norm for women in Bangladesh and thus, discouraged for honest disclosure. There are mixed findings of smoking behaviors in other studies; while some studies documented that migrant and urban groups are more likely to smoke than rural groups (44–46), other studies showed reverse findings (38, 42). Alcohol consumption shows similar gender difference as for smoking behavior but it was more prevalent in the urban group than migrants. In the Peru migrant study heavy drinking in the past year was similar between the urban, migrant, and rural groups (44) whereas in the pool analyses of the WHO-SAGE there were significantly lower alcohol consumption in urban and migrant groups compared to rural residents (42).

The present study showed that women were more vulnerable than men to mental health disorders. The prevalence of probable cases was the highest among migrant women (35.9%) and the lowest was found among urban men (16.8%). Any kind of migration poses stress on migrants which can compromise their mental health (47). One of the underlying reasons is the disruption to family life such as loss of support from family and social networks. Being separated from family and friends and facing stress in the adjustment process can increase vulnerability to psychological illness (47, 48). Findings from a longitudinal study of rural-to-urban migrants in Thailand reported that migrants were mentally healthier upon arrival, nevertheless, their mental health deteriorated within 2–4 years after migration (49). The Peru migrant study also reported that the prevalence of mental disorders was higher in migrants (38%) compared to non-migrants in urban areas (33%), however, these analyses were not stratified by gender (50). The opposite scenario was documented in a study of Chinese rural-to-urban migrant workers where migrants were mentally healthier than their urban non-migrant counterparts (51). These two possible directions of trends in mental health migration process and that many different factors may be involved including cultural differences between rural and urban settings in particular countries and the level of acculturation of migrants.

### 4.2. CVD risk factors and the role of SES

The present study showed a positive association between SES, particularly strong by household wealth, and most of the CVD risk factors among both migrant and urban groups. Among men, there were negative strong associations between smoking behavior and household wealth and educational attainment for urban and migrant alike. Similarly, alcohol/illicit drug use decreased with higher education and wealth.

Poor mental health particularly depression has been established as an independent risk factor for CVD (52). We found that mental health disorder was higher in lower SES men and women compared to high SES individuals, for urban residence and migrants alike. This may indicate less financial and personal resources (e.g., education) to overcome hardship in the lower SES group, whereas the high SES group are more able to seek mental care or are equipped with educational resources to combat distress and hardship. The 2010 Bangladesh NCD risk factor survey also reported that diabetes, hypertension, physical inactivity and obesity increased with socio-economic achievements, though no data on mental health was collected to support our finding above (53). This SES pattern may vary by place of residence as indicated by another study in Bangladesh which demonstrated that although hypertension, diabetes, and overweight/obesity were more prevalent among the richest, after stratification by place of residence, a high prevalence for these conditions was found among the wealthy in urban areas and the poor in rural areas (54). This paradoxical relationship between social gradients and behavioral, weight-related and metabolic risks indicates that an epidemiological transition may be ongoing (55).

### 4.3. Duration of urban stay and acculturation to urban life

In this study, we took length of urban residence as a proxy measure of acculturation, and weight gain as the immediate consequence of adoption of unhealthy lifestyle, which are the results of the acculturation process. Another study has reported that unhealthy weight gain among migrants significantly increases after 10–15 years of migration (22), in line with our findings. A cross-sectional migrant study on CVD risk factors and duration of urban residence in India, also showed that adiposity increased rapidly within one decade after moving to an urban area, whereas other CVD risk factors such as blood pressure, and diabetes developed progressively up to the fourth decade (12). Whether most of the weight gain occurs in the earlier years of migration or creeps slowly over time, would be interesting to know, in particular to inform the potential urgency of early intervention. A longitudinal study in Thailand reported that hypertension and hyperlipidemia were associated with urbanization and recent migrants (within the past 4 years) and long-term urban dwellers had higher risk than rural dwellers (56). This is also supported by the Peru Migrant longitudinal study that followed migrants for 5 years after migration and reported that migrant and urban groups had an 8-to-9.5 times higher risk of developing obesity than the rural group (57). The increase of overweight and obesity after migration can be explained by sudden lifestyle changes and adaptation, which is related to the consumption of abundant high-calorie fatty foods, low consumption of fruits and vegetables and a decrease in physical activity (2, 22). The association between unhealthy diet and acculturation has been documented before (58, 59).

In this study, cigarette smoking increased with time spent in urban living, whereas bidi smoking decreased with length of urban living. Given bidi is more prevalent in rural areas, this practice has dissolved over time among these migrants suggesting that acculturation happened over time in this study groups. These findings are comparable to study in China (60), Peru (44), Tanzania (61), and Indonesia (48) where smoking rates were positively associated with duration of urban stay. In the case of alcohol/illicit drug use, while our results showed a significant positive trend, in Peru (44) and Tanzania (61) non-significant increases in alcohol consumption over time were observed.

Length of stay in urban areas was associated with an increased risk of migrants' psychological distress in here as in China, where a mental health score increased soon after migration but decreased with the course of time (49). In contrast, two studies evaluating rural-to-urban migration and mental health showed the opposite relationship; one study in India reported that migrants who lived in an urban area for more than 15 years had a lower mental health disorders (60) and another in Indonesia where recent migrants who lived in urban areas for < 3 years were more at risk of mental health disorders particularly in females (48), however the detrimental effect of migration on mental health was minimal among those who moved with their family (48). The present study reported that the risk of mental health disorders significantly increased with time since migration only in women and that women who moved to urban areas for work or study were at two times higher risk of having hypertension than those who moved for familial reasons. This may be explained by the social support either from family, like in the Indonesian study (48) or interaction with the settlers of origin at the urban destination through a strong network as in Peru (50) where migrants maintained a strong network with origin settlers but it dissolved over time due to acculturation to the urban culture and they then became isolated. As women migrant were found more vulnerable in this study, further in depth study could reveal the underlying reason.

### 4.4. Strength and limitations

Our study has several strengths. Analyses were conducted on a large representative dataset of all urban areas in Bangladesh. This is also the first study in Bangladesh to compare the distribution of risk factors between migrants and non-migrants and to examine the prevalence of CVD risk factors in migrants by duration of urban residence. Further, the findings are robust given adjustment for socio-economic factors that were assessed in the UHS.

Our study, however, was not exempt from limitations. The main limitation is the cross-sectional design that cannot examine causality. While a longitudinal study design is ideal to examine how CVD risk evolves over time in relation to migration, such type of study is difficult due to feasibility, especially where a population registry is not available to assist with identification and tracing of migrants in the total population. Secondly, we compared the health status of migrants with non-migrant urban dwellers; however, a more appropriate approach would be to compare migrants with the benchmark i.e., similar people of origin. We also could not consider temporary and circular migration. Thirdly, in this study multiple adults were selected from each household, which may have diluted the differences of CVD risk factors if adults from the same household were classified in two different study groups given they shared similar environment and diet. In addition, measurement of diabetes by capillary blood glucose is not a diagnostic measure (unlike the OGTT) and diabetes and hypertension was only measured among those aged >35 years and excluded District Municipalities, hence limiting the generalisability. Finally, as there is hardly any nationally representative data on CVD risk factors among migrants, we had to use comparatively old dataset. However, we get an understanding of the situation through this study and this situation is supposed to get worse over time. National surveys on health should consider inclusion of both factors to monitor trends of CVD risk among migrants.

## 5. Conclusion

The findings of our study highlight the distribution of CVD risk among rural-to-urban migrants, according to duration since migration. Migrants had high proportion of CVD risk factors which were influenced by education, wealth and duration of urban stay. This study showed that migrants had high proportion of CVD risk factors which reached the level of urban non-migrants and these risk factors appear to increase with duration of residence. As acculturation is an obvious phenomena of migration, and in this case acculturation results in development of risk factors, strategies to change this course of events in both urban non-migrants and rural-to-urban migrants at early stage must be developed.

## Data availability statement

The original contributions presented in the study are included in the article/Supplementary material, further inquiries can be directed to the corresponding author.

## Ethics statement

The studies involving human participants were reviewed and approved by Western Sydney University Human Research Ethics Committee (HREC # H11056). The patients/participants provided their written informed consent to participate in this study.

## Author contributions

SM conceptualized the study, compiled the data, carried out the data analysis and interpretation, and drafted the manuscript. FS critically reviewed and edited the manuscript. DM conceptualized the study, supported data analysis, and interpretation and drafting manuscript. All authors contributed to the article and approved the submitted version.
