# Supplementary material for: Rural-to-urban migration, socio-economic status and cardiovascular diseases risk factors among Bangladeshi adults: A nationwide population based survey
Source: Front Public Health. 2023 Apr 6;11:860927. doi: 10.3389/fpubh.2023.860927 (PMC10116049; doi:10.3389/fpubh.2023.860927)
Supplement: Supplementary file 1 [file Table_1.docx]

# **Supplementary File 1**

**Table** **S1: Role of SES explaining the association between migration status and CVD risk factors**

| **OR** | **SES** | **Obesity** | **hypertension** | **Diabetes** | **Mental Health Disorder** | **Cigarette smoking** | **Bidi smoking** | **Alcohol/illicit drug use** |  |
| --- | --- | --- | --- | --- | --- | --- | --- | --- | --- |
| **Men** | | | | | | | | | |
| **Crude** | Urban | Ref | Ref | Ref | Ref | Ref | Ref | Ref |  |
|  | Migrant | 0.66 (0.59-0.74) | 0.88 (0.70-1.11) | 0.98 (0.68-1.44) | 1.17 (1.07-1.27) | 0.97 (0.90-1.03) | 1.99 (1.76-2.27) | 0.61 (0.55-0.68) |  |
| **Stratum specific** | **Education** |  |  |  |  |  |  |  |  |
|  | Illiterate to Primary | 0.64 (0.51-0.79) | 0.88 (0.62-1.24) | 1.05 (0.49-2.26) | 1.05 (0.93-1.78) | 1.11 (0.99-1.22) | 1.60 (1.38-1.85) | 0.62 (0.54-0.72) |  |
|  | High school and above | 0.83 (0.72-0.95) | 1.02 (0.75-1.39) | 1.17 (0.75-1.81) | 1.10 (0.96-1.26) | 0.75 (0.68-0.82) | 1.79 (1.31-2.45) | 0.53 (0.45-0.63) |  |
| **Adjusted** | M-H OR | 0.77 (0.68-0.87) | 0.95 (0.76-1.20) | 1.14 (0.78-1.67) | 1.07 (0.98-1.17) | 0.89 (0.84-0.96) | 1.64 (1.44-1.86) | 0.58 (0.52-0.65) |  |
|  | % Change | 16% | 8% | 16% | 8% | 7% | 18% | 5% |  |
| **Stratum specific** | **HH Wealth Quintile** |  |  |  |  |  |  |  |  |
|  | Q1 & Q2 | 0.71 (0.55-0.93) | 1.32 (0.82-2.12) | 1.41 (0.49-4.02) | 0.99 (0.88-1.13) | 0.94 (0.84-1.04) | 1.65 (1.42-1.91) | 0.59 (0.51-0.69) |  |
|  | Q3 &Q4 | 0.79 (0.66-0.93) | 0.88 (0.62-1.24) | 0.98 (0.53-1.80) | 1.11 (0.96-1.30) | 0.92 (0.83-1.03) | 1.17 (0.86-1.59) | 0.55 (0.45-0.66) |  |
|  | Q5 | 1.10 (0.88-1.38) | 0.99 (0.64-1.52) | 1.21 (0.69-2.13) | 1.09 (0.83-1.43) | 0.71 (0.60-0.85) | 2.39 (0.53-10.71) | 0.59 (0.44-0.79) |  |
| **Adjusted** | M-H OR | 0.85 (0.75-0.96) | 1.01 (0.80-1.27) | 1.13 (0.77-1.67) | 1.05 (0.96-1.14) | 0.89 (0.84-0.96) | 1.55 (1.36-1.77) | 0.58 (0.52-0.64) |  |
|  | % Change | 28% | 14% | 15% | 10% | 8% | 22% | 6% |  |
| **Women** | | | | | | | | | |
| **Crude** | Urban | Ref | Ref | Ref | Ref | Ref | Ref | Ref |  |
|  | Migrant | 0.66 (0.60-0.73) | 0.77 (0.61-0.97) | 0.73 (0.51-1.07) | 1.14 (1.06-1.22) |  | - |  |  |
| **Stratum specific** | **Education** |  |  |  |  |  |  |  |  |
|  | Illiterate to Primary | 0.63 (0.55-0.73) | 0.82 (0.61-1.09) | 0.75 (0.44-1.28) | 1.03 (0.94-1.12) |  | - |  |  |
|  | High school and above | 0.89 (0.77-1.03) | 0.93 (0.83-1.04) | 0.92 (0.53-1.59) | 0.97 (0.86-1.09) |  |  |  |  |
| **Adjusted** | M-H OR | 0.74 (0.67-0.82) | 0.82 (0.65-1.04) | 0.83 (0.57-1.22) | 1.01 (0.94-1.08) |  |  |  |  |
|  | % Change | 12% | 6% | 13% | 11% |  | - |  |  |
| **Stratum specific** | **HH Wealth Quintile** |  |  |  |  |  | | |  |
|  | Q1 & Q2 | 0.70 (0.58-0.85) | 0.84 (0.52-1.35) | 3.21 (0.40-25.71) | 1.01 (0.91-1.21) |  |  |  |  |
|  | Q3 &Q4 | 0.93 (0.79-110) | 1.14 (0.78-1.66) | 0.95 (0.49-1.83) | 1.03 (0.92-1.16) |  | - |  |  |
|  | Q5 | 1.01 (0.81-1.27) | 0.81 (0.53-1.23) | 0.94 (0.56-1.57) | 1.08 (0.89-1.31) |  |  |  |  |
| **Adjusted** | M-H OR | 0.87 (0.78-0.97) | 0.94 (0.74-1.20) | 1.01 (0.68-1.49) | 1.03 (0.96-1.10) |  |  |  |  |
|  | % Change | 32% | 44% | 37% | 10% |  | - |  |  |
